# Pre-conditioning with PQQ during pregnancy alleviates LPS-induced placental damage and improves the fetal survival and growth in mice

**DOI:** 10.3389/fendo.2025.1617026

**Published:** 2025-07-10

**Authors:** Yongli Han, Yan Zhu, Xiaohui Lu, Tingting Fan, Yina Yin, Pengfei Liu, Xiuliang Dai, Hongbin Xu

**Affiliations:** ^1^ Department of Nursing, Changzhou Hygiene Vocational Technology College, Changzhou, Jiangsu, China; ^2^ Obstetrics and Gynecology Department, The Second People’s Hospital of Changzhou, the Third Affiliated Hospital of Nanjing Medical University, Changzhou, China; ^3^ The Center for Reproductive Medicine, Changzhou Maternal and Child Health Care Hospital, Changzhou Medical Center, Nanjing Medical University, Changzhou, Jiangsu, China; ^4^ The Department of Animal Center, Kebiao Medical Testing Center, Changzhou, Jiangsu, China

**Keywords:** intrauterine infection, fetal health, pyrroloquinoline quinone, placental damage, pre-conditioning

## Abstract

**Background:**

Intrauterine infection is a major cause of preterm birth, fetal demise, and growth restriction. Placental damage resulting from such infections plays a central role in mediating these adverse outcomes. Pyrroloquinoline quinone (PQQ) is a naturally occurring nutrient known for its antioxidant, anti-inflammatory, and mitochondrial-supporting properties. This study aimed to investigate whether pre-conditioning with PQQ during pregnancy could mitigate adverse effects induced by lipopolysaccharide (LPS)-mediated inflammation in mice.

**Methods:**

Pregnant mice were randomly assigned to three groups: control, LPS, and LPS + PQQ. On gestational day (GD) 16.5, mice in the LPS groups were intraperitoneally injected with either a single dose of 3 μg/mouse (moderate inflammation) or two doses of 3ug/mouse (severe inflammation) of LPS. In the LPS + PQQ group, PQQ was administered daily from GD 0.5. Outcomes assessed included labor time, fetal survival, fetal and placental weights. Placental structure, vascular networks, inflammation, oxidative stress, and gene expression profiles were evaluated using H&E staining, immunohistochemistry, Prussian blue staining, and RNA sequencing.

**Results:**

Pre-conditioning with PQQ significantly alleviated LPS-induced fetal demise and reduced fetal and placental growth. PQQ also improved placental morphology, restored vascular integrity, and normalized aberrant gene expression profiles. Furthermore, PQQ treatment markedly reduced placental inflammation and oxidative stress in mice exposed to moderate LPS. However, under high-dose LPS conditions, PQQ failed to confer significant protective effects.

**Conclusion:**

Our findings suggest that Pre-conditioning with PQQ during pregnancy can protect against inflammation-induced placental damage and improve fetal survival and growth under moderate inflammatory conditions. This study provides compelling proof-of-concept that PQQ buffers the placenta against maternal systemic inflammatory insults. However, its efficacy appears limited in the context of severe inflammation.

## 1 Introduction

Intrauterine infections during pregnancy affects approximately 1-4% of all births in the US, can lead to various maternal and neonatal complications, including preterm labor, sepsis, cerebral palsy, and even stillbirth (1, 2). Currently, anti-infection strategies, such as broad-spectrum intravenous antibiotics, are used to control bacterial infections. However, adjuvant therapies that can preserve placental function and fetal health are still lacking. Additionally, for pregnant women at high risk of intrauterine infection, such as those with a history of previous infections, preventive medicines are still not available.

The placenta, serving as the crucial connection between the mother and the developing fetus, facilitates nutrient and oxygen exchange, waste removal, and hormone production to support pregnancy. It can, however, be easily affected by intrauterine infections. Intrauterine infection can lead to intensified and widespread inflammation and oxidative stress, thereby damaging the structure and function of the placenta (3). As a result, the placenta’s ability to exchange oxygen and nutrients becomes compromised, leading to adverse fetal outcomes such as growth restriction, preterm premature rupture of membranes, fetal brain damage, and even stillbirth (3). It has been demonstrated that targeting inflammation or oxidative stress could alleviate infection induced placental damage, thereby improving fetus outcomes (4–6).

Pyrroloquinoline quinone (PQQ), initially identified as a bacterial co-factor (7), is also enriched in human breast milk (8). Recent studies have demonstrated that PQQ is essential for health in animals, including humans (9). PQQ deficiency has been shown to severely impact the development of mice, leading to growth retardation, immune dysfunction, and reproductive disorders (10). In contrast, PQQ supplementation can help prevent the progression of various diseases, including alkylating agent-induced ovarian dysfunction (11, 12). Administering PQQ during pregnancy has been shown to protect obese offspring from developing nonalcoholic fatty liver disease and to prevent developmental programming of microbial dysbiosis (13, 14). The health benefits of PQQ are closely linked to its antioxidant and anti-inflammatory properties (9). Additionally, PQQ is known as a mitochondrial enhancer, promoting mitochondrial biogenesis (15). Due to its safety, commercial PQQ products are available for improving human health for healthy adults, but not pregnant and lactating women (16). This is due to the limited data available on the effects of PQQ supplementation during pregnancy. Therefore, the use of PQQ in pregnant women is still limited and require more research. Given these, we aim to investigate whether dietary intake of PQQ could protect the placenta from damage in the context of intrauterine infection

In the present study, we used lipopolysaccharide (LPS) to establish a model of intrauterine infection in pregnant mice, mimicking bacterial infection. PQQ was supplemented through the diet starting on day 0.5 after pregnancy confirmation and continued until delivery or sacrifice. We then assessed the effects of PQQ treatment on fetal outcomes, placental structure, placental vessels network, gene expression profiles in placenta, inflammation and oxidative stress markers in placenta. In addition, we also tested whether PQQ can protect the fetal outcomes and placenta structure in response to high dose LPS.

## 2 Materials and methods

### 2.1 Mice and treatment

Two-to three-month-old male and female BALB/c mice were purchased from Cavance Animal Company and housed in a specific pathogen-free (SPF) animal facility with ad libitum access to food and water, maintained under a 12-hour light/dark cycle. One female mouse was mated with one male mouse at 6 PM. The presence of a clear vaginal plug in the female mouse examined the following morning indicated successful mating, marking that day as gestational day (GD) 0.5. The pregnant mice were randomly divided into three groups: the Control group, the LPS group, and the PQQ+LPS group. PQQ, purchased from Shandong Weifang Company, was incorporated into the mouse’s diet at a dosage of 5 mg/kg. The food containing PQQ was provided to the mice in the PQQ+LPS group starting from GD 0.5 and continued until delivery or sacrifice. On GD 16.5, to establish a moderate infection model, LPS (3 ug per mouse) was administered via intraperitoneal injection once. To establish a severe infection model, the same dose of LPS (3ug per mouse) was injected intraperitoneally twice at a 3-hour interval. Some pregnant mice in each group were maintained until delivery, while the remaining mice in each group were sacrificed on GD 17.5, the weight of placenta and fetus were recorded, placentas were collected for experimental use. The detailed study design was described in **Supplementary Figure 1**.

### 2.2 Histology and immunohistochemistry

The fresh placentas were fixed in 4% paraformaldehyde (PFA) solution for 48 hours. Following fixation, the placental tissues were dehydrated, cleared, and embedded in paraffin. Paraffin-embedded placentas were sectioned at a thickness of 5 µm. The sections were then dewaxed and rehydrated for subsequent histological and immunohistochemical staining.

For hematoxylin and eosin (HE) staining, the sections were stained sequentially with hematoxylin followed by eosin.

For Prussian blue staining, a commercial Prussian blue staining kit (BP-DL161, SenBeiJia Biological Technology, China) was used.

For immunohistochemistry, the sections were subjected to antigen retrieval by boiling in Tris-EDTA solution for 10 mins. Endogenous peroxidase activity was blocked by incubating the sections with 3% H_2_O_2_. The sections were then incubated with 10% donkey serum for blocking for 1 hour. The sections were then incubated overnight at 4C with primary antibodies, including anti-CK7 antibody (17513-1-AP, Proteintech, China), anti-CD31 antibody (#77699, Cell Signaling Technology, China), anti-IL6 antibody (GB11117, Servicebio, China), anti-p65 antibody (GB11142, Servicebio, China), anti-8-OHdG antibody (sc-393871, Santa Cruz, China). After washing off the primary antibodies, the sections were incubated with horseradish peroxidase (HRP)-conjugated goat anti-rabbit (A0208, Beyotime Biotechnology, China) or anti-mouse (A0216, Beyotime Biotechnology, China) secondary antibodies for 1 hour. The sections were visualized using DAB staining and counterstained with hematoxylin, followed by rinsing with running water to restore the blue color. Following dehydration and clearing, the slides were mounted, and photographs were taken.

### 2.3 Quantification of histochemistry and *immunohistochemistry*

For quantification of CK-7 positive area for each group, five representative images of CK7-positive staining from 5 placentas and 5 pregnant mice for each group, were used for measuring the CK7-positive areas by using Image J. The CK7-positive area in the control group was used as the reference for comparison.

Quantification of vascular parameters, including vessel area, number of branch points, number of end points and mean lacunarity, five representative CD31-immunostained images from 5 placentas and 5 pregnant mice for each group, were processed by using Angio tool to measure vascular parameters (17). The parameters in Control group were set as reference for comparison.

For quantification of Prussian blue clusters, number of Prussian blue clusters per section were evaluated by visual observation using five representative images from 5 placentas and 5 pregnant mice for each group.

For quantification of IL6 intensity, five representative images of immunohistochemistry staining of IL6 from 5 placentas and 5 pregnant mice for each group, were used for measuring the IL6 intensity by using Image J. The IL6 intensity in Control group was set as reference for comparison.

For quantification of p65 and 8-OHdG nuclear positive cells, five representative images of immunohistochemistry staining of p65 and 8-OHdG from 5 placentas and 5 pregnant mice for each group, were used for measuring p65 and 8-OHdG nuclear positive cells by using Image J. The percentage of p65 and 8-OHdG nuclear positive cells were calculated as positive cells to the whole cells.

### 2.4 RNA-sequencing and analysis

Fresh placentas (n=3 in each group) were immediately immersed in liquid nitrogen, and stored in -80 °C for later use.

#### 2.4.1 RNA isolation and library preparation

Total RNA was extracted using the TRIzol reagent (Invitrogen, CA, USA) according to the manufacturer’s protocol. RNA purity and concentration were assessed using the NanoDrop 2000 spectrophotometer (Thermo Scientific, USA). RNA integrity was evaluated using the Agilent 2100 Bioanalyzer (Agilent Technologies, Santa Clara, CA, USA). Library construction was performed using the VAHTS Universal V10 RNA-seq Library Prep Kit (Premixed Version), following the manufacturer’s instructions. Transcriptome sequencing and analysis were conducted by OE Biotech Co., Ltd. (Shanghai, China).

#### 2.4.2 RNA sequencing and differentially expressed genes analysis

The libraries were sequenced on an Illumina NovaSeq 6000 platform, generating 150 bp paired-end reads. For each sample, 39.04 to 48.59 million raw reads were obtained. Raw FASTQ files were initially processed using fastp (18), and low-quality reads were removed to obtain clean reads. After filtering, 38.35 to 47.66 million clean reads per sample were retained for downstream analysis. Clean reads were aligned to the mouse reference genome using HISAT2 (19). Gene expression levels were quantified as fragments per kilobase of transcript per million mapped reads (FPKM) (20), and read counts were generated using HTSeq-count (21). Principal component analysis (PCA) was conducted in R (v3.2.0) to evaluate the biological replicates. Differential expression analysis was performed using DESeq2 (22), with significantly differentially expressed genes (DEGs) defined as those with a Q value < 0.05 and a fold change > 2 or < 0.5. The gene expression levels between groups were provided in **Supplementary Data**. Hierarchical clustering of DEGs was conducted using R (v3.2.0) to visualize gene expression patterns across different groups and samples. Gene Ontology (GO) (23) and KEGG (24) enrichment analyses were performed to identify significantly enriched terms using the hypergeometric distribution in R (v3.2.0). Bubble plots were generated in R to visualize the enrichment results.

### 2.5 Statistical analysis

Data are expressed as mean ± SD. Statistical significance among groups was determined using one-way ANOVA, followed by Tukey’s multiple comparisons test. GraphPad Software (Version 11) was used for statistical analysis. A p-value of < 0.05 was considered statistically significant.

## 3 Results

### 3.1 Pre-conditioning with PQQ improved fetal survival in pregnant mice treated with LPS

After the injection of LPS, pregnant mice were observed every hour until parturition occurred in the control group for recording labor time and viability of fetuses. Preterm birth and decreased fetal survival are the primary outcomes of intrauterine infection (25) (26, 27). Initially, we used two dosages (3ug/mouse) of LPS to establish intrauterine infection model. Our results showed that all pregnant mice treated with high-dose LPS delivered within 24 hours, and no viable fetuses were observed (**Supplementary Figures 2A, B**). PQQ treatment had no effect on the incidence of preterm birth or fetal survival under these conditions (**Supplementary Figures 2A, B**). In addition, some fetuses were delivered enclosed within an intact amniotic sac (**Supplementary Figure 2C**). HE staining revealed profound structural changes in the placental labyrinth, including disrupted architecture, enlarged vessels, and accumulation of blood cells, observed in both the LPS and PQQ groups (**Supplementary Figure 2D**). These findings indicate that PQQ did not confer protective effects in the context of severe infection during pregnancy.

**Figure 1:**
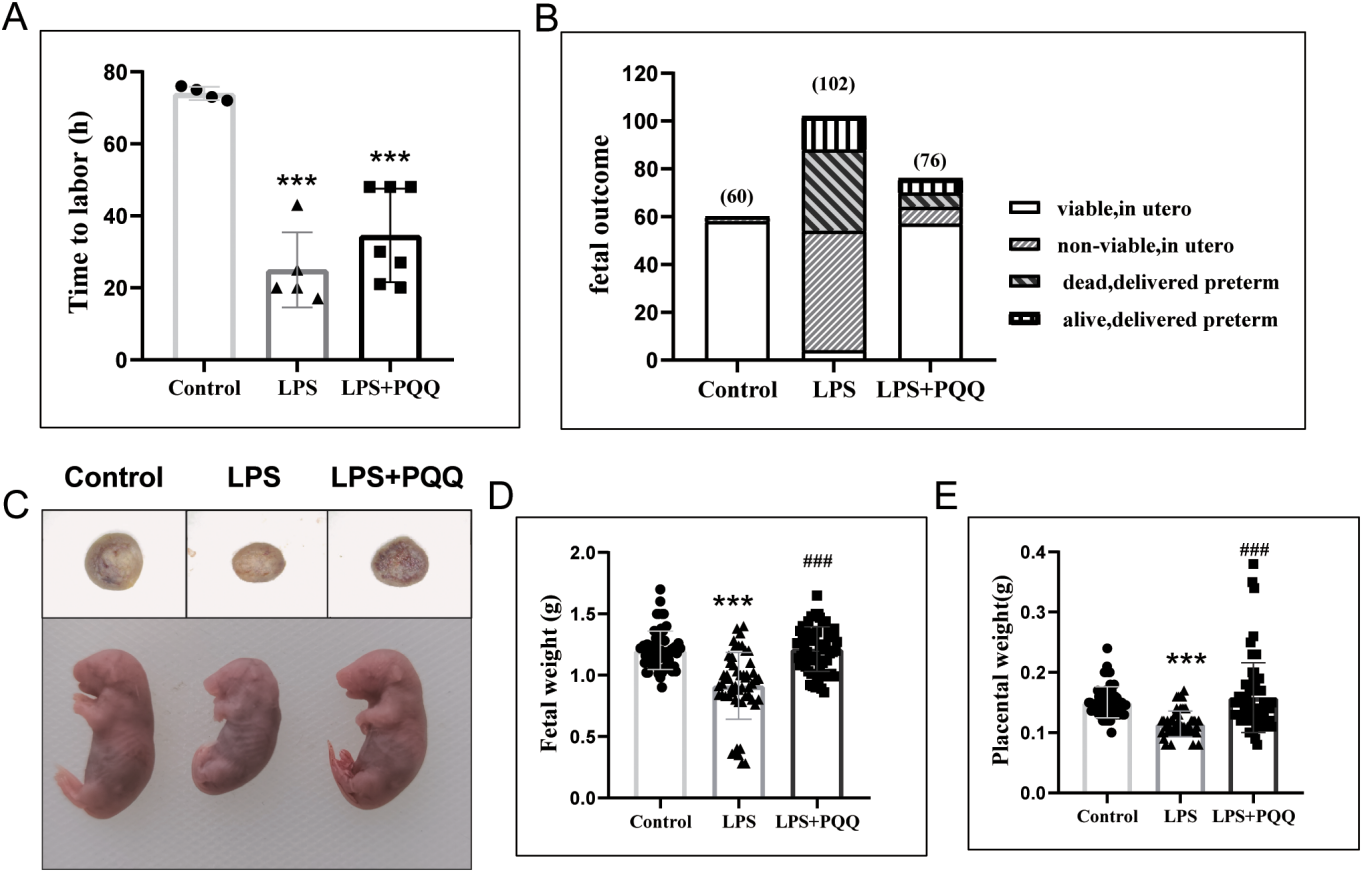
Pre-conditioning with PQQ improves fetal survival in pregnant mice treated with LPS. (**A**) Mice received either normal saline or LPS (3 µg/mouse), and the time to labor after treatment was recorded. n=4 pregnant mice for control group, n=5 pregnant mice for LPS group and n=7 pregnant mice for PQQ group. **(B)** Twenty-four hours post-treatment, for mice that had delivered, the number of surviving and deceased fetuses was recorded; for still-pregnant mice, the animals were sacrificed, and fetuses were collected to assess survival *in utero*. Twenty-four hours post-treatment, the still-pregnant mice were sacrificed, n=5 pregnant mice in Control group, n=10 pregnant mice in LPS group, and n=7 pregnant mice in PQQ group. **(C)** The representative image of the placenta and fetus among groups, **(D)** Fetal weight and **(E)** placental weight were assessed. n=60 fetuses in control group from 5 pregnant mice, n=54 in LPS group from 5 pregnant mice, and n= 64 in PQQ group from 6 pregnant mice. Statistical significance: ***p < 0.001 vs. Control group; ^###^p < 0.001 vs. LPS group.

We speculated that a high dose of LPS induced overwhelming inflammation, which may have masked the effect of PQQ. Therefore, we used a single dose of LPS to establish the intrauterine infection model. As expected, LPS treatment at gestational day (GD) 16.5 induced preterm birth in mice, decreased fetal survival, and resulted in smaller fetal and placental size and weight (**Figures 1A–E**). Compared to pregnant mice in the LPS group, mice in the PQQ group had a longer time to labor following LPS treatment, although the difference was not statistically significant (**Figure 1A**). Here, preterm delivery was defined as delivery occurring before gestational day GD 19. Further analysis showed that in the Control group, the percentage of preterm birth was 0/4 (0%), in the LPS group it was 5/5 (100%), and in the PQQ group it was 4/7 (57.14%), indicating that PQQ supplementation inhibited LPS-induced preterm birth in some degree. Strikingly, fetal survival in the PQQ group was significantly higher compared to the LPS group, and fetal and placental size and weight were significantly increased (**Figures 1B–E**). These results collectively demonstrated a protective effect of PQQ in alleviating LPS-induced complications in pregnant mice.

### 3.2 Pre-conditioning with PQQ improves placental structure in pregnant mice treated with LPS

**Figure 2:**
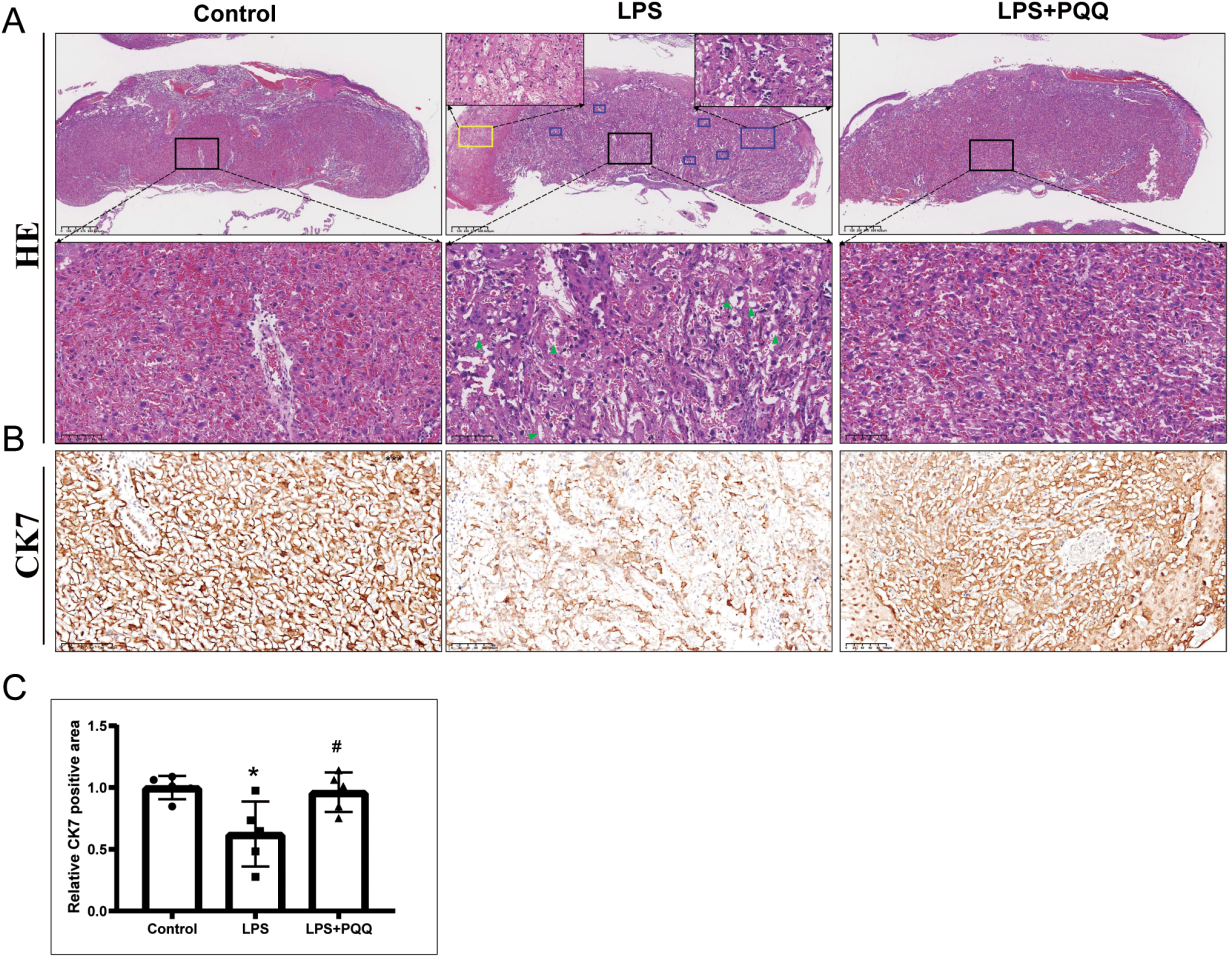
Pre-conditioning with PQQ improves placental structure in pregnant mice treated with LPS. Twenty-four hours post-treatment, for still-pregnant mice, the placentas were collected for further analysis. **(A)** HE staining displayed the placenta structure. The lower magnified images displayed regions of labyrinth layer. Blue rectangle indicates region of placental calcification, yellow rectangle indicates region of placental infarction. Green triangle indicates absence of red blood cells. For each group, N = 5 placentas from 5 pregnant mice. **(B)** Immunohistochemistry staining of CK7 in placenta labyrinth layer. For each group, N = 5 placentas from 5 pregnant mice. **(C)** Quantification of CK7 positive areas. *p < 0.05 vs. Control group; #p < 0.05 vs. LPS group.

We then examined placental morphological changes among groups. We found that LPS treatment induced placental calcification and infarction, disrupted tissue architecture, and caused the absence of red blood cells in the labyrinth region (**Figure 2A**). These abnormalities were barely observed in the PQQ-treated group (**Figure 2A**). CK7 is primarily expressed in trophoblasts within the mouse placental labyrinth, where it supports vascular development and maternal-fetal exchange (28). We found that the expression of CK7 was significantly reduced in the placenta following LPS treatment when compared to the control (**Figures 2B, C**). However, PQQ treatment significantly increased CK7 expression (**Figures 2B, C**), indicating that PQQ treatment protected the placenta against LPS-induced damage to trophoblasts. These results collectively demonstrated that PQQ treatment can largely prevent the structural abnormalities of the placenta caused by LPS.

### 3.3 Pre-conditioning with PQQ improved vascular condition and vessel instability in placenta treated with LPS

**Figure 3:**
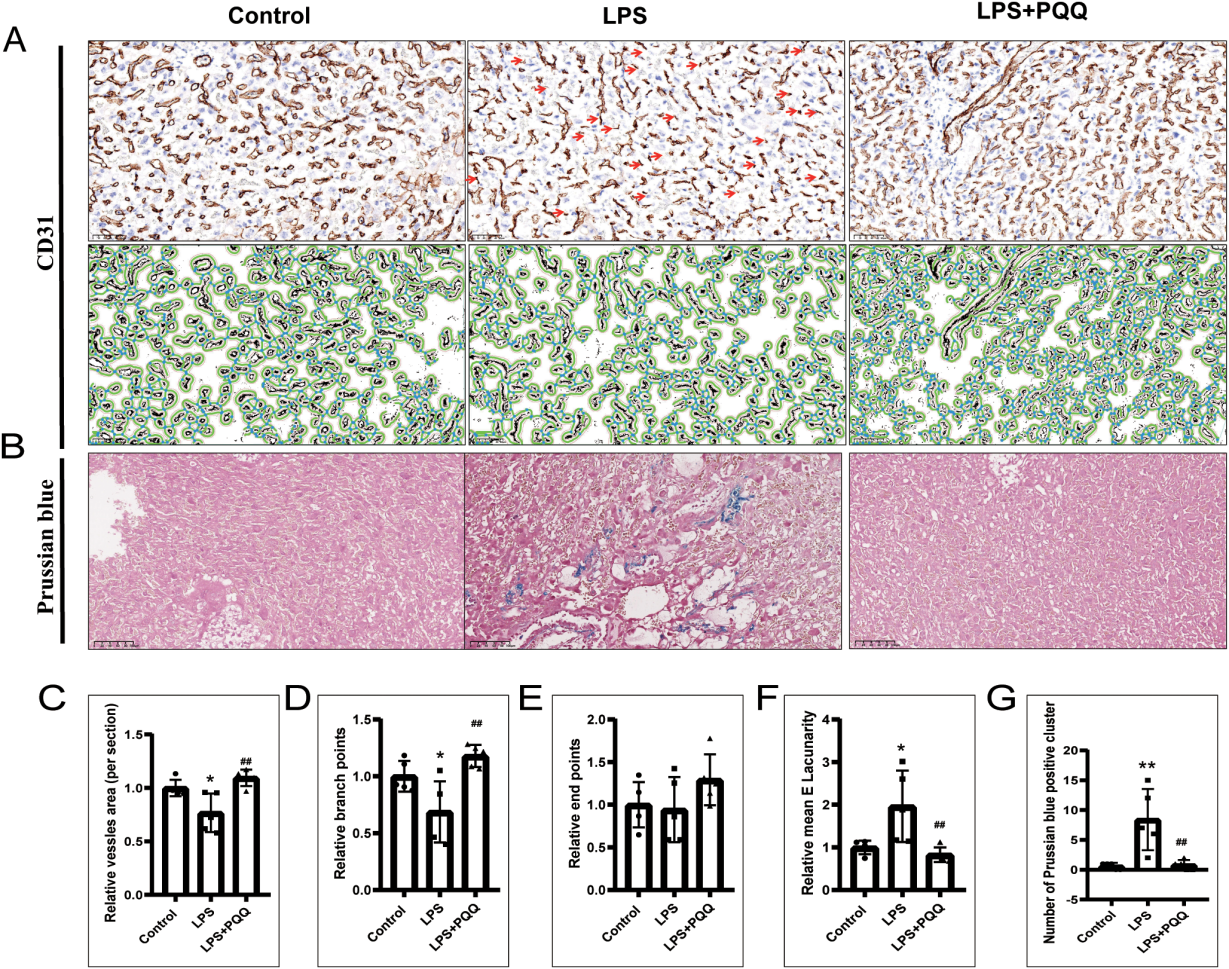
PQQ Pre-conditioning improves vascular condition and reduces vessel instability in placentas of LPS-treated mice. Twenty-four hours after treatment, placentas from still-pregnant mice were collected for further analysis. **(A)** Upper panels: Immunohistochemical staining of CD31 in the placental labyrinth layer. The red arrow indicates prominent, line-like CD31-positive staining, suggestive of vessel regression. Lower panels: The corresponding images were analyzed using AngioTool based on CD31 immunohistochemical staining. Green lines outline placental microvessels; blue dots indicate branch points. For each group, N = 5 placentas from 5 pregnant mice. **(B)** Prussian blue staining of placental sections. For each group, N = 5 placentas from 5 pregnant mice. Quantification of vascular parameters: **(C)** Vessel area, **(D)** Number of branch points, **(E)** Number of end points, **(F)** Mean lacunarity. **(G)** Number of Prussian blue clusters. *p < 0.05, **p < 0.01 vs. Control group; ^##^p < 0.01 vs. LPS group.

The vessels in the placental labyrinth play a critical role in facilitating efficient maternal-fetal exchange of oxygen, nutrients, and waste products, thereby ensuring proper fetal development. It is known that acute inflammation can lead to vascular dysfunction (29). Our results showed that LPS treatment induced prominent line-like CD31-positive staining, indicating vessel regression (**Figure 3A**). Quantification of CD31 immunohistochemistry by Angio tool revealed that LPS also reduced the overall vessel area and the number of branch points, while increasing the mean E lacunarity value reflecting greater heterogeneity, irregularity, and gaps in vessel architecture (**Figures 3A, C, D, F**). However, LPS treatment had no significant effect on the number of endpoints (**Figures 3A, E**). In addition, LPS treatment significantly increased the number of Prussian blue-positive cell clusters in the placenta, indicative of vascular instability (**Figures 3B, G**). Compared to the LPS group, the PQQ-treated group exhibited more normal vascular morphology, with increased overall vessel area and branch points, and reduced mean E lacunarity and Prussian blue-positive clusters (**Figures 3A–G**). Collectively, these findings demonstrated that PQQ treatment largely rescues the vascular abnormalities induced by LPS.

### 3.4 Pre-conditioning with PQQ largely corrected the gene expression alternations in placenta treated with LPS

**Figure 4:**
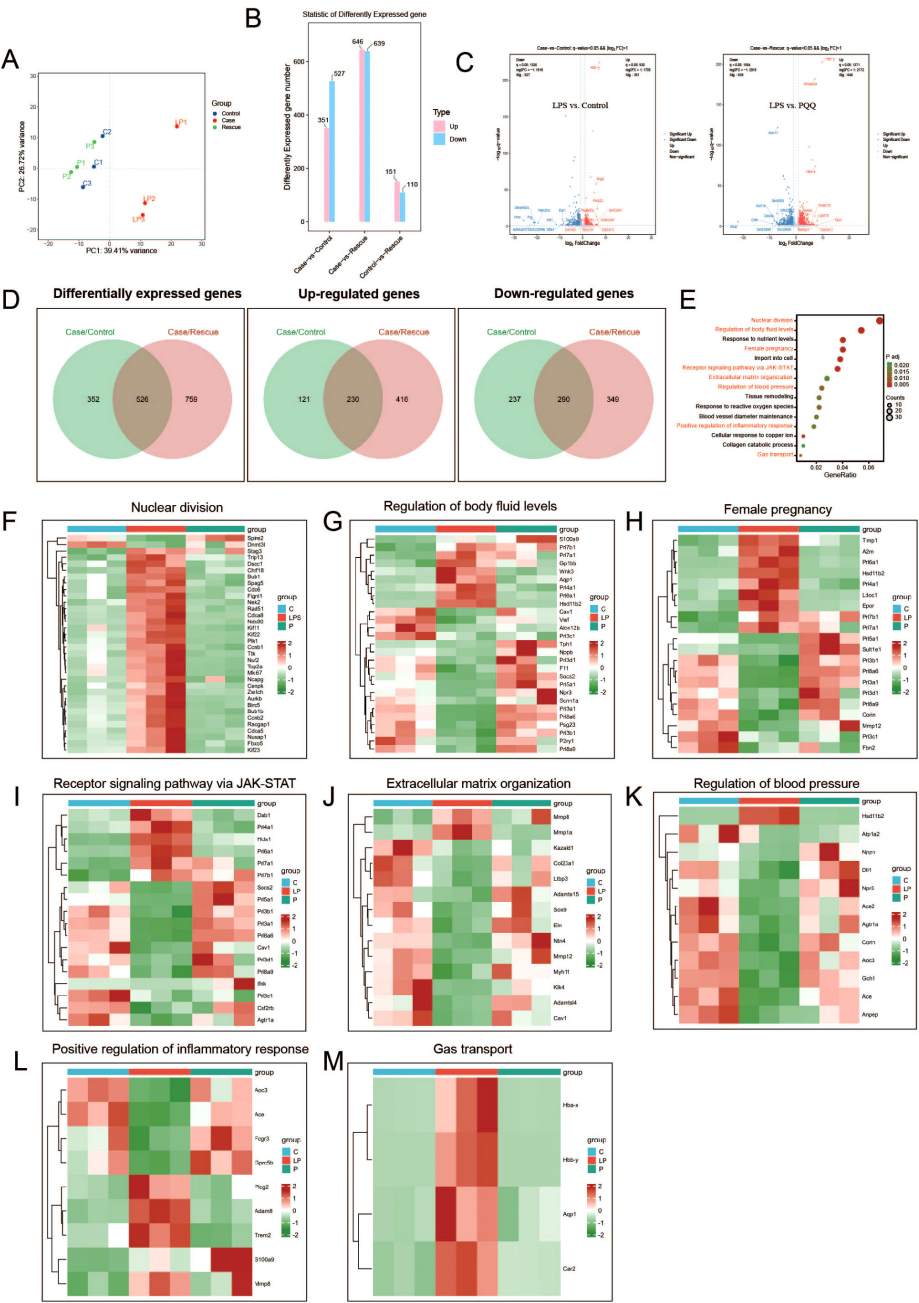
PQQ Pre-conditioning largely corrects gene expression alterations in placentas of LPS-treated mice. Twenty-four hours after treatment, placentas from still-pregnant mice were collected for further analysis. **(A)** Principal component analysis (PCA) of RNA sequencing samples. **(B)** Differentially expressed genes between the Case (LPS) and Control **(C)**, Case (LPS) and Rescue (PQQ), and Control **(C)** and Rescue (PQQ) groups. **(C)** Volcano plot showing differentially expressed genes between the LPS and Control, and LPS and PQQ groups. **(D)** Number of common up-regulated and down-regulated genes in the Control and PQQ groups compared to the LPS group. **(E)** Bubble chart showing KEGG analysis of the top biological processes (LPS vs. Control). Gene expression involved in specific biological processes: **(F)** Nuclear division, **(G)** Regulation of body fluid levels, **(H)** Female pregnancy, **(I)** Receptor signaling pathways via JAK-STAT, **(J)** Extracellular matrix organization, **(K)** Regulation of blood pressure, **(L)** Positive regulation of inflammatory response and **(M)** Gas transport, among the Control, LPS, and PQQ groups. N=3 placentas from 3 pregnant mice for each group.

To comprehensively investigate the protective effect of PQQ on the placenta at the gene expression level, we performed RNA sequencing on samples from the Control, LPS, and PQQ (Rescue) groups. Principal component analysis (PCA) of the RNA-seq data revealed that samples from the Control and PQQ groups clustered closely together, whereas samples from the LPS group were clearly separated, indicating distinct transcriptomic changes induced by LPS (**Figure 4A**). Compared to the Control group, the LPS group exhibited 351 upregulated and 527 downregulated genes, while the PQQ group showed 151 upregulated and 110 downregulated genes (**Figure 4B**). When compared to the LPS group, the PQQ group showed 646 upregulated and 639 downregulated genes (**Figure 4B**). Volcano plots highlighted the most significantly differentially expressed genes in both LPS vs. Control and LPS vs. PQQ comparisons (**Figure 4C**). Further analysis revealed a total of 526 differentially expressed genes shared between the LPS vs. Control and LPS vs. PQQ comparisons (**Figure 4D**). Among these, 230 genes were commonly upregulated and 290 genes were commonly downregulated (**Figure 4D**). KEGG pathway analysis of differentially expressed genes in the LPS vs. Control comparison revealed multiple significantly altered biological processes, we showed 15 representative biological processes here (**Figure 4E**). From these, eight pathways closely related to infection or pregnancy were selected for further analysis. We then examined the expression patterns of genes involved in nuclear division, regulation of body fluid levels, female pregnancy, receptor signaling pathway via JAK-STAT, extracellular matrix organization, regulation of blood pressure, positive regulation of inflammatory response, and gas transport across the three groups. In all eight pathways, gene expression patterns in the PQQ group were similar to those in the Control group and clearly different from the LPS group (**Figures 4F–M**). Collectively, these transcriptomic data further supported that PQQ treatment mitigates LPS-induced placental damage.

### 3.5 PQQ Pre-conditioning improves inflammation and oxidative stress status in placentas of LPS-treated mice

**Figure 5:**
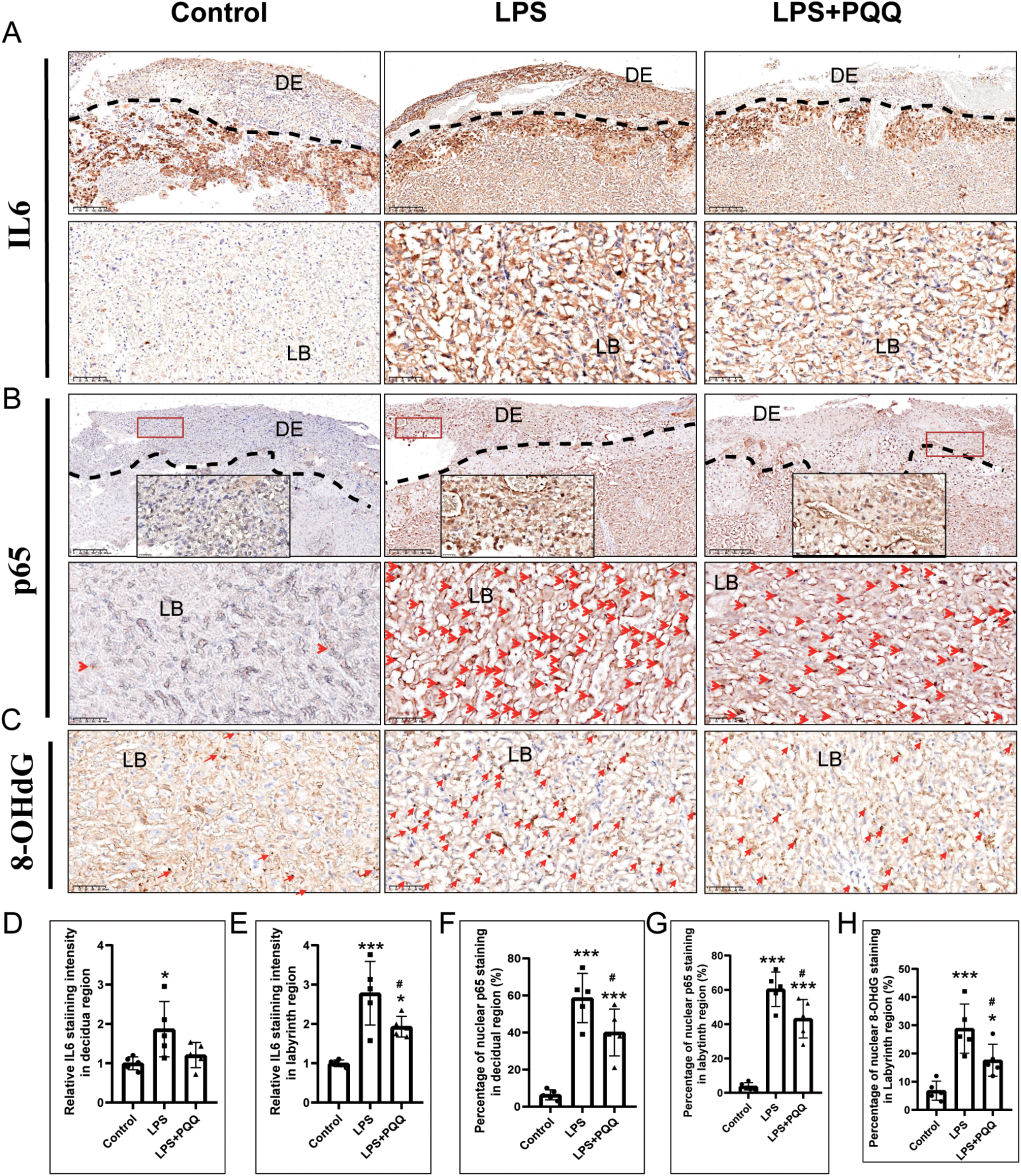
PQQ Pre-conditioning improves inflammation and oxidative stress status in placentas of LPS-treated mice. Twenty-four hours after treatment, placentas from still-pregnant mice were collected for further analysis. **(A)** Immunohistochemical staining of IL-6 in the placenta. N=5 placentas from 5 pregnant mice for each group. The upper panel indicates the DE region located above the dashed line. The lower panel indicates LB. **(B)** Immunohistochemical staining of p65 in the placenta. The upper panel indicates the DE located above the dashed line. The area within the red rectangle is shown at higher magnification in the inset. The lower panel indicates LB. Red arrows indicate p65-positive staining. N=5 placentas from 5 pregnant mice for each group. **(C)** Immunohistochemical staining of 8-OHdG in the DE region of the placenta. N=5 placentas from 5 pregnant mice for each group. Quantification of IL-6 expression in the **(D)** decidual area and **(E)** labyrinth layer. Quantification of p65-positive cells in the **(F)** decidual area and **(G)** labyrinth layer. **(H)** Quantification of 8-OHdG-positive cells in the labyrinth layer. *p < 0.05, ***p < 0.001 vs. Control group; # p < 0.05 vs. LPS group. DE, Decidual area; LB, labyrinth layer.

Inflammation and oxidative stress are key mediators of LPS-induced placental damage. We showed that LPS treatment significantly increased the expression of IL-6 and the nuclear expression of p65 in the placental decidual and labyrinth regions (**Figures 5A, B, D–G**). Compared to the LPS group, PQQ treatment significantly decreased the expression of IL-6 and nuclear p65 in both the decidual and labyrinth regions of the placenta (**Figures 5A, B, D–G**). 8-OHdG, a marker of oxidative stress and DNA oxidation, was significantly increased in the placental labyrinth region following LPS treatment, compared to the control group (**Figures 5C, H**). However, PQQ treatment significantly reduced the percentage of 8-OHdG-positive cells in the placental labyrinth region (**Figures 5C, H**). Together, these results suggested that PQQ treatment alleviates placental inflammation and oxidative stress induced by LPS.

## 4 Discussion

In this study, we demonstrated that Pre-conditioning with PQQ significantly alleviated LPS-induced complications in a murine model of intrauterine infection, including fetal demise, as well as reduced fetal and placental growth. Our findings highlight the potential of PQQ as a protective agent during pregnancy under conditions of mild to moderate intrauterine inflammation. Although it appears that PQQ prolonged the time to labor following LPS treatment, the difference was not statistically significant. It is known that the fetal membrane plays a more direct role in the initiation of preterm birth (30, 31). Therefore, the protective effect of PQQ on the fetal membrane may not be as very pronounced. Additionally, the protective efficacy of PQQ is limited under severe inflammatory conditions.

The placenta is a crucial organ that supports pregnancy by facilitating nutrient and gas exchange between the mother and fetus, producing hormones essential for fetal development and maternal adaptation, and providing immune protection (32). Indeed, the placenta can be directly damaged by infection during pregnancy. It has been reported that maternal LPS exposure leads to placental mal-perfusion, increased fetal vessel resistance, and histological changes in the placental structure, including infarctions, calcifications, and increased intervillous spaces (33–35). The placental alterations caused by infection are considered a primary cause of fetal and neonatal morbidity and mortality (36). In the present study, we also observed that LPS treatment significantly induced changes in placental structure, including infarctions and calcifications. CK7 predominantly mark trophoblast lineages within the labyrinth region of the mouse placenta (28). These cells contribute to the development and maintenance of the labyrinth’s vascular architecture, thereby facilitating efficient exchange of nutrients, gases, and waste products between the mother and fetus. A previous study has showed that conditional mutation of Hand1 significantly reduced CK7-positive syncytial trophoblasts, leading to fewer fetal vessels, misorganization of maternal blood spaces, and impaired labyrinth structure (28). Consistently, we also observed a decreased CK7 positive cells in LPS-treated placenta. However, all these placental abnormalities caused by LPS were largely rescued by Pre-conditioning with PQQ. These results demonstrate that PQQ treatment improve placental structure in pregnant mice treated with LPS.

Placental vasculature is essential for supporting the development of embryos. Previous studies have shown that systemic maternal infection and subsequent inflammation can disrupt placental vasculogenesis and angiogenesis (3). Placental vascularization, vascular remodeling, and oxygen transport are severely affected by maternal infection (37–40). Consistent with these findings, in the present study, we observed massive irregular and short, line-shaped CD31-positive staining, indicating vessel regression in LPS-treated placentas. Moreover, LPS treatment also induced a decrease in vessel area, branching points, and led to a heterogeneous and disordered vascular network. It has been reported that inflammation causes endothelial cell injury and disrupts vascular integrity, thereby inducing bleeding (41–43). Consistently, we also observed Prussian blue staining in LPS-treated placentas, further supporting the instability of the vessels. These vascular abnormalities in the placenta were largely corrected by Pre-conditioning with PQQ. This is consistent with previous studies reporting a protective effect of PQQ on endothelial cells from high glucose- or doxorubicin-induced damage (44, 45). Therefore, PQQ treatment improves vascular damage in placenta caused by LPS.

Our transcriptional data revealed that the transcriptional landscape of PQQ-treated placentas resembled that of unchallenged controls, in stark contrast to the divergent gene profile observed in the LPS group. Further analysis showed that PQQ treatment normalized gene expression patterns across key biological processes, including nuclear division, regulation of body fluid levels, female pregnancy, receptor signaling pathway via JAK-STAT, extracellular matrix organization, regulation of blood pressure, positive regulation of inflammatory response, and gas transport. This raises a critical question: did PQQ directly modulate the molecules involved in such a broad range of biological processes, or are the transcriptional changes observed a consequence of PQQ’s ability to mitigate LPS-induced damage? Theoretically, it is unlikely that PQQ directly targets such a broad range of molecules. For instances, we found that LPS treatment significantly upregulated genes involved in cell proliferation and gas transport. We speculate that, moderate infection induced placental damage may trigger inflammation-induced proliferation and increased expression of oxygen-related genes, serving as a defensive mechanism to repair the damaged structures and enhance oxygen-carrying efficiency. Interestingly, PQQ treatment suppressed the expression of all these genes, which may suggest an inhibition of the defensive system. However, if PQQ were indeed suppressing the defensive system, it would be expected to exacerbate LPS-induced placental damage. Clearly, this is not the case. On the contrary, PQQ treatment improves fetal outcomes and alleviates placental damage. Therefore, the transcriptional changes likely reflect an overall improvement in LPS-induced placental injury by PQQ, providing compelling evidence for the protective role of PQQ. However, this prompted us to further investigate the mechanisms by which PQQ prevents LPS-induced placental damage.

It is well established that LPS-induced placental damage is primarily mediated by inflammation. Besides the direct damage caused by inflammation, it has been reported that inflammation-induced oxidative stress also contributes to infection-induced placental damage (4, 46, 47). Therefore, it is likely that PQQ treatment limits the spread of inflammation and alleviates LPS-induced oxidative stress at an early stage, thereby preventing subsequent placental damage. In addition, mountains of studies have demonstrated the protective effect of PQQ against various diseases via its anti-inflammatory and antioxidant properties (9). As expected, our data revealed significant suppression of IL-6 expression, p65 nuclear translocation, and 8-OHdG accumulation in PQQ-treated placentas. Additionally, a recent study has highlighted the crucial role of mitochondrial dysfunction in LPS-induced inflammation and damage (48). PQQ is known as a mitochondrial optimizer (15), playing a powerful role in promoting mitochondrial biogenesis. Therefore, we speculate that the protective role of PQQ against intrauterine infection may also be attributed to its protective effect in preserving mitochondrial function.

The mechanisms by which PQQ combats inflammation have been increasingly explored in recent years. It has been reported that PQQ can inhibit p65 nuclear translocation and MAPK activation, thereby suppressing inflammatory responses (49, 50). Min et al. has demonstrated that PQQ alleviates allergic airway inflammation in mice by modulating the immune microenvironment and regulating the JAK-STAT signaling pathway (51). In addition, Wu et al. identified CUL3 as a key effector through which PQQ exerts its anti-inflammatory effects (52). However, despite these findings, the precise molecular mechanisms by which PQQ, as a nutritional factor, regulates such diverse signaling pathways remain to be fully elucidated and warrant further investigation.

In the present study, the timing of PQQ administration, prior to LPS insult, may be a critical determinant of its efficacy. This raises an important biological and translational question: Is PQQ acting as a Pre-conditioning agent, “priming” the placenta and maternal immune system to a more tolerant or resilient state? Pre-conditioning strategies, well-studied in ischemia-reperfusion injury and cardioprotection (53, 54), have only recently been considered in obstetrics (55). PQQ may represent a molecular bridge between metabolic Pre-conditioning and placental protection.

The present study holds clinical significance. As a nutritional factor, PQQ is recommended for dietary supplementation in humans. Clinical studies have shown that supplementation with PQQ offers multifaceted benefits, including improvement of age-related mild cognitive decline (56, 57), enhancement of brain function in both younger and older individuals (58), increased cerebral blood flow and oxygen metabolism (59), and decreased LDL cholesterol levels (60). Clinical studies have also indicated the antioxidant, anti-inflammatory, and mitochondrial biogenesis-promoting effects of PQQ (61, 62). The use of PQQ is quite safe, as evidenced by the no-observed-adverse-effect-level (NOAEL) of 100 mg/kg body weight per day from a 90-day repeated-dose oral toxicity study with BioPQQ™ (16). However, it remains uncertain whether dietary supplementation with PQQ is appropriate for pregnant women. Animal studies, however, have shown beneficial effects of PQQ supplementation, including increased cardiomyocyte endowment in spontaneous IUGR guinea pigs (63),improvements in offspring liver bioactive lipid profiles and protection against the development of adult NAFL in mice (64, 65), amelioration of L-NAME-induced preeclampsia-like symptoms in rats (66), mitigation of MK-801-induced schizophrenia-like behaviors in mice (67), and improved intestinal health of offspring in mice (68, 69). Therefore, PQQ holds promise for future clinical applications in the treatment of intrauterine infections.

Despite these promising results, the effective protective effects of PQQ were not observed in mice exposed to a high dose of LPS. All fetuses were non-viable regardless of PQQ treatment, and placental damage was severe. Our findings suggest a threshold model in which the degree of inflammatory burden dictates the reversibility of placental pathology. Once structural collapse is triggered, antioxidant intervention may be insufficient. Clinically, this underscores the urgent need for early diagnostic tools to identify pregnancies at risk before irreversible damage occurs.

Another important consideration is that in the present study, we used LPS to mimic infection, rather than live bacteria. This model differs from actual bacterial infection, as it does not account for the expansion of bacterial populations. It is important to recognize that the *in vivo* anti-inflammatory effects of PQQ alone may not be sufficient. In a true infection, the host’s inflammatory response plays a critical role in bacterial clearance. We observed that PQQ may alleviate the inflammatory response; however, if bacterial expansion is not controlled, the protective effects of PQQ may ultimately be lost. For this reason, combining PQQ with antibacterial strategies may be necessary for the effective treatment of intrauterine infections. Although a recent study showed that PQQ exhibited notable antibacterial activity against Gram-positive and -negative bacteria (70). Future work should explore the translational potential of PQQ in higher-order models, its pharmacokinetics during pregnancy, and its combinatorial effects with antimicrobial agents. Additionally, future studies should investigate whether PQQ acts primarily through modulation of mitochondrial function, suppression of innate immune sensors, or preservation of endothelial-trophoblast crosstalk. Delineating the precise molecular targets of PQQ may yield novel strategies to safeguard pregnancy against inflammatory disruption.

## 5 Conclusion

In summary, our preliminary data indicate that Pre-conditioning with PQQ during pregnancy can protect against inflammation-induced placental damage and improve fetal survival and growth under moderate inflammatory conditions in mice. This study provides compelling proof-of-concept that PQQ buffers the placenta against maternal systemic inflammatory insults. However, its efficacy appears limited in the context of severe inflammation.

## Data Availability

The original RNA-Seq data in this article have been deposited in the NCBI SRA under the accession number PRJNA1283190 (SRR34283230–SRR34283238). The data can be accessed at: https://www.ncbi.nlm.nih.gov/sra/PRJNA1283190. Other data underlying this article will be made available upon reasonable request to the corresponding author.

## References

[1] Tita ATN WWJCiP A. Diagnosis and management of clinical chorioamnionitis. Clin Perinatol. (2010) 37:339–54. doi: 10.1016/j.clp.2010.02.003, PMID: 20569811 PMC3008318

[2] Jung E Romero R Suksai M Gotsch F Chaemsaithong P Erez O. Clinical chorioamnionitis at term: definition, pathogenesis, microbiology, diagnosis, and treatment. Am J Obstet Gynecol. (2024) 230:S807–S40. doi: 10.1016/j.ajog.2023.02.002, PMID: 38233317 PMC11288098

[3] Weckman AM Ngai M Wright J McDonald CR Kain KC. The impact of infection in pregnancy on placental vascular development and adverse birth outcomes. Front Microbiol. (2019) 10:1924. doi: 10.3389/fmicb.2019.01924, PMID: 31507551 PMC6713994

[4] Paintlia MK Paintlia AS Singh AK Singh I. Attenuation of lipopolysaccharide-induced inflammatory response and phospholipids metabolism at the feto-maternal interface by N-acetyl cysteine. Pediatr Res. (2008) 64:334–9. doi: 10.1203/PDR.0b013e318181e07c, PMID: 18552708 PMC2967178

[5] Chen YH Yu Z Fu L Wang H Chen X Zhang C. Vitamin D3 inhibits lipopolysaccharide-induced placental inflammation through reinforcing interaction between vitamin D receptor and nuclear factor kappa B P65 subunit. Sci Rep. (2015) 5:10871. doi: 10.1038/srep10871, PMID: 26065916 PMC4464284

[6] Zhang J Luo X Huang C Pei Z Xiao H Luo X. Erythropoietin prevents lps-induced preterm birth and increases offspring survival. Am J Reprod Immunol. (2020) 84:e13283. doi: 10.1111/aji.13283, PMID: 32506750 PMC7507205

[7] Hauge JG. Glucose dehydrogenase of bacterium anitratum: an enzyme with a novel prosthetic group. J Biol Chem. (1964) 239:3630–9. doi: 10.1016/S0021-9258(18)91183-X, PMID: 14257587

[8] Mitchell AE Jones AD Mercer RS Rucker RB. Characterization of pyrroloquinoline quinone amino acid derivatives by electrospray ionization mass spectrometry and detection in human milk. Anal Biochem. (1999) 269:317–25. doi: 10.1006/abio.1999.4039, PMID: 10222004

[9] Yan T Nisar MF Hu X Chang J Wang Y Wu Y. Pyrroloquinoline quinone (Pqq): its impact on human health and potential benefits: pqq: human health impacts and benefits. Curr Res Food Sci. (2024) 9:100889. doi: 10.1016/j.crfs.2024.100889, PMID: 39513102 PMC11541945

[10] Steinberg FM Gershwin ME Rucker RB. Dietary pyrroloquinoline quinone: growth and immune response in balb/C mice. J Nutr. (1994) 124:744–53. doi: 10.1093/jn/124.5.744, PMID: 8169668

[11] Jonscher KR Chowanadisai W Rucker RB. Pyrroloquinoline-quinone is more than an antioxidant: A vitamin-like accessory factor important in health and disease prevention. Biomolecules. (2021) 11:1441. doi: 10.3390/biom11101441, PMID: 34680074 PMC8533503

[12] Dai X Yi X Wang Y Xia W Tao J Wu J. Pqq dietary supplementation prevents alkylating agent-induced ovarian dysfunction in mice. Front Endocrinol (Lausanne). (2022) 13:781404. doi: 10.3389/fendo.2022.781404, PMID: 35340329 PMC8948422

[13] Friedman JE Dobrinskikh E Alfonso-Garcia A Fast A Janssen RC Soderborg TK. Pyrroloquinoline quinone prevents developmental programming of microbial dysbiosis and macrophage polarization to attenuate liver fibrosis in offspring of obese mice. Hepatol Commun. (2018) 2:313–28. doi: 10.1002/hep4.1139, PMID: 29507905 PMC5831029

[14] Steinberg F Stites TE Anderson P Storms D Chan I Eghbali S. Pyrroloquinoline quinone improves growth and reproductive performance in mice fed chemically defined diets. Exp Biol Med (Maywood). (2003) 228:160–6. doi: 10.1177/153537020322800205, PMID: 12563022

[15] Charrier D Cerullo G Carpenito R Vindigni V Bassetto F Simoni L. Metabolic and biochemical effects of pyrroloquinoline quinone (Pqq) on inflammation and mitochondrial dysfunction: potential health benefits in obesity and future perspectives. Antioxidants (Basel)Epub 2024/09/28.(2024) 1322:45. doi: 10.3390/antiox13091027, PMID: 39334686 PMC11429417

[16] Turck D Bresson J-L Burlingame B Dean T Fairweather-Tait S Heinonen M. afety of pyrroloquinoline quinone disodium salt as a novel food pursuant to regulation (Ec) no 258/97. EFSA J. (2017) 15:e05058. doi: 10.2903/j.efsa.2017.5058, PMID: 32625350 PMC7010138

[17] Zudaire E Gambardella L Kurcz C Vermeren S. A computational tool for quantitative analysis of vascular networks. . PloS One. (2011) 6:e27385. doi: 10.1371/journal.pone.0027385, PMID: 22110636 PMC3217985

[18] Chen S Zhou Y Chen Y Gu J. Fastp: an ultra-fast all-in-one fastq preprocessor. Bioinformatics. (2018) 34:i884–i90. doi: 10.1093/bioinformatics/bty560, PMID: 30423086 PMC6129281

[19] Kim D Langmead B Salzberg SL. Hisat: A fast spliced aligner with low memory requirements. Nat Methods. (2015) 12:357–60. doi: 10.1038/nmeth.3317, PMID: 25751142 PMC4655817

[20] Roberts A Trapnell C Donaghey J Rinn JL Pachter L. Improving rna-seq expression estimates by correcting for fragment bias. Genome Biol. (2011) 12:R22. doi: 10.1186/gb-2011-12-3-r22, PMID: 21410973 PMC3129672

[21] Anders S Pyl PT Huber W. Htseq–a python framework to work with high-throughput sequencing data. Bioinformatics. (2015) 31:166–9. doi: 10.1093/bioinformatics/btu638, PMID: 25260700 PMC4287950

[22] Love MI Huber W Anders S. Moderated estimation of fold change and dispersion for rna-seq data with deseq2. Genome Biol. (2014) 15:550. doi: 10.1186/s13059-014-0550-8, PMID: 25516281 PMC4302049

[23] The Gene Ontology C. The gene ontology resource: 20 years and still going strong. Nucleic Acids Res. (2019) 47:D330–D8. doi: 10.1093/nar/gky1055, PMID: 30395331 PMC6323945

[24] Kanehisa M Araki M Goto S Hattori M Hirakawa M Itoh M. Kegg for linking genomes to life and the environment. Nucleic Acids Res. (2008) 36:D480–4. doi: 10.1093/nar/gkm882, PMID: 18077471 PMC2238879

[25] Daskalakis G Psarris A Koutras A Fasoulakis Z Prokopakis I Varthaliti A. Maternal infection and preterm birth: from molecular basis to clinical implications. Children (Basel). (2023) 10:907. doi: 10.3390/children10050907, PMID: 37238455 PMC10217143

[26] Hudalla H Karenberg K Kuon RJ Poschl J TsChada R Frommhold D. Lps-induced maternal inflammation promotes fetal leukocyte recruitment and prenatal organ infiltration in mice. Pediatr Res. (2018) 84:757–64. doi: 10.1038/s41390-018-0030-z, PMID: 30135596

[27] Cotechini T Hopman WJ Graham CH. Inflammation-induced fetal growth restriction in rats is associated with altered placental morphometrics. Placenta. (2014) 35:575–81. doi: 10.1016/j.placenta.2014.05.002, PMID: 24927914

[28] Courtney JA Wilson RL Cnota J Jones HN. Conditional mutation of hand1 in the mouse placenta disrupts placental vascular development resulting in fetal loss in both early and late pregnancy. Int J Mol Sci. (2021) 22:9532. doi: 10.3390/ijms22179532, PMID: 34502440 PMC8431056

[29] Zanoli L Briet M Empana JP Cunha PG Maki-Petaja KM Protogerou AD. Vascular consequences of inflammation: A position statement from the esh working group on vascular structure and function and the artery society. J Hypertens. (2020) 38:1682–98. doi: 10.1097/HJH.0000000000002508, PMID: 32649623 PMC7610698

[30] Pan J Tian X Huang H Zhong N. Proteomic study of fetal membrane: inflammation-triggered proteolysis of extracellular matrix may present a pathogenic pathway for spontaneous preterm birth. Front Physiol. (2020) 11:800. doi: 10.3389/fphys.2020.00800, PMID: 32792973 PMC7386131

[31] Menon R Richardson LS Lappas M. Fetal membrane architecture, aging and inflammation in pregnancy and parturition. Placenta. (2019) 79:40–5. doi: 10.1016/j.placenta.2018.11.003, PMID: 30454905 PMC7041999

[32] Burton GJ Fowden AL. The placenta: A multifaceted, transient organ. Philos Trans R Soc Lond B Biol Sci. (2015) 370:20140066. doi: 10.1098/rstb.2014.0066, PMID: 25602070 PMC4305167

[33] Fricke EM Elgin TG Gong H Reese J Gibson-Corley KN Weiss RM. Lipopolysaccharide-Induced Maternal Inflammation Induces Direct Placental Injury without Alteration in Placental Blood Flow and Induces a Secondary Fetal Intestinal Injury That Persists into Adulthood. Am J Reprod Immunol. (2018) 79:e12816. doi: 10.1111/aji.12816, PMID: 29369434 PMC5908742

[34] Eloundou SN Lee J Wu D Lei J Feller MC Ozen M. Placental malperfusion in response to intrauterine inflammation and its connection to fetal sequelae. PloS One. (2019) 14:e0214951. doi: 10.1371/journal.pone.0214951, PMID: 30943260 PMC6447225

[35] Dijkstra F Jozwiak M De Matteo R Duncan J Hale N Harding R. Erythropoietin ameliorates damage to the placenta and fetal liver induced by exposure to lipopolysaccharide. Placenta. (2010) 31:282–8. doi: 10.1016/j.placenta.2009.12.028, PMID: 20106521

[36] Sato Y. Inflammatory lesions in placental pathology. J Obstet Gynaecol Res. (2022) 48:58–65. doi: 10.1111/jog.14932, PMID: 34729867

[37] Szaba FM Tighe M Kummer LW Lanzer KG Ward JM Lanthier P. Zika virus infection in immunocompetent pregnant mice causes fetal damage and placental pathology in the absence of fetal infection. PloS Pathog. (2018) 14:e1006994. doi: 10.1371/journal.ppat.1006994, PMID: 29634758 PMC5909921

[38] Tabata T Petitt M Fang-Hoover J Rivera J Nozawa N Shiboski S. Cytomegalovirus impairs cytotrophoblast-induced lymphangiogenesis and vascular remodeling in an *in vivo* human placentation model. Am J Pathol. (2012) 181:1540–59. doi: 10.1016/j.ajpath.2012.08.003, PMID: 22959908 PMC3483806

[39] Zhang Y Sheng Z Chen Q Zhou A Cao J Xue F. Neutrophil infiltration leads to fetal growth restriction by impairing the placental vasculature in denv-infected pregnant mice. EBioMedicine. (2023) 95:104739. doi: 10.1016/j.ebiom.2023.104739, PMID: 37544202 PMC10432184

[40] McDonald CR Cahill LS Gamble JL Elphinstone R Gazdzinski LM Zhong KJY. Malaria in pregnancy alters L-arginine bioavailability and placental vascular development. Sci Transl Med. (2018) 10:10(431). doi: 10.1126/scitranslmed.aan6007, PMID: 29514999 PMC6510298

[41] van Hinsbergh VW. Endothelium–role in regulation of coagulation and inflammation. Semin Immunopathol. (2012) 34:93–106. doi: 10.1007/s00281-011-0285-5, PMID: 21845431 PMC3233666

[42] Saitoh H Sakaguchi M Miruno F Muramatsu N Ito N Tadokoro K. Histopathological analysis of lipopolysaccharide-induced liver inflammation and thrombus formation in mice: the protective effects of aspirin. Curr Issues Mol Biol. (2024) 46:14291–303. doi: 10.3390/cimb46120856, PMID: 39727984 PMC11674652

[43] McMullan RR McAuley DF O’Kane CM Silversides JA. Vascular leak in sepsis: physiological basis and potential therapeutic advances. Crit Care. (2024) 28:97. doi: 10.1186/s13054-024-04875-6, PMID: 38521954 PMC10961003

[44] Wang Z Chen GQ Yu GP Liu CJ. Pyrroloquinoline quinone protects mouse brain endothelial cells from high glucose-induced damage *in vitro* . Acta Pharmacol Sin. (2014) 35:1402–10. doi: 10.1038/aps.2014.4, PMID: 25283505 PMC4220070

[45] Jiang C Jiang L Li Q Liu X Zhang T Yang G. Pyrroloquinoline quinine ameliorates doxorubicin-induced autophagy-dependent apoptosis via lysosomal-mitochondrial axis in vascular endothelial cells. Toxicology. (2019) 425:152238. doi: 10.1016/j.tox.2019.152238, PMID: 31226464

[46] Vieira LD Farias JS de Queiroz DB Cabral EV Lima-Filho MM Sant’Helena BRM. Oxidative stress induced by prenatal lps leads to endothelial dysfunction and renal haemodynamic changes through angiotensin ii/nadph oxidase pathway: prevention by early treatment with alpha-tocopherol. Biochim Biophys Acta Mol Basis Dis. (2018) 1864:3577–87. doi: 10.1016/j.bbadis.2018.09.019, PMID: 30254014

[47] Park S Shin J Bae J Han D Park SR Shin J. Sirt1 Alleviates Lps-Induced Il-1beta Production by Suppressing Nlrp3 Inflammasome Activation and Ros Production in Trophoblasts. Cells. (2020) 9:728. doi: 10.3390/cells9030728, PMID: 32188057 PMC7140679

[48] Purandare N Kunji Y Xi Y Romero R Gomez-Lopez N Fribley A. Lipopolysaccharide induces placental mitochondrial dysfunction in murine and human systems by reducing mnrr1 levels via a tlr4-independent pathway. iScience. (2022) 25:105342. doi: 10.1016/j.isci.2022.105342, PMID: 36339251 PMC9633742

[49] Yang C Yu L Kong L Ma R Zhang J Zhu Q. Pyrroloquinoline quinone (Pqq) inhibits lipopolysaccharide induced inflammation in part via downregulated nf-kappab and P38/jnk activation in microglial and attenuates microglia activation in lipopolysaccharide treatment mice. PloS One. (2014) 9:e109502. doi: 10.1371/journal.pone.0109502, PMID: 25314304 PMC4196908

[50] Liu Z Sun C Tao R Xu X Xu L Cheng H. Pyrroloquinoline quinone decelerates rheumatoid arthritis progression by inhibiting inflammatory responses and joint destruction via modulating nf-kappab and mapk pathways. Inflammation. (2016) 39:248–56. doi: 10.1007/s10753-015-0245-7, PMID: 26319019

[51] Min Z Zhou J Mao R Cui B Cheng Y Chen Z. Pyrroloquinoline quinone administration alleviates allergic airway inflammation in mice by regulating the jak-stat signaling pathway. Mediators Inflammation. (2022) 2022:1267841. doi: 10.1155/2022/1267841, PMID: 36345503 PMC9637035

[52] Wu Y Zhao M Lin Z. Pyrroloquinoline quinone (Pqq) alleviated sepsis-induced acute liver injury, inflammation, oxidative stress and cell apoptosis by downregulating cul3 expression. Bioengineered. (2021) 12:2459–68. doi: 10.1080/21655979.2021.1935136, PMID: 34227919 PMC8806920

[53] Yang X Cohen MV Downey JM. Mechanism of cardioprotection by early ischemic preconditioning. Cardiovasc Drugs Ther. (2010) 24:225–34. doi: 10.1007/s10557-010-6236-x, PMID: 20505987 PMC2932886

[54] Iliodromitis EK Lazou A Kremastinos DT. Ischemic preconditioning: protection against myocardial necrosis and apoptosis. Vasc Health Risk Manag. (2007) 3:629–37., PMID: 18078014 PMC2291307

[55] Hao D He C Ma B Lankford L Reynaga L Farmer DL. Hypoxic preconditioning enhances survival and proangiogenic capacity of human first trimester chorionic villus-derived mesenchymal stem cells for fetal tissue engineering. Stem Cells Int. (2019) 2019:9695239. doi: 10.1155/2019/9695239, PMID: 31781252 PMC6874947

[56] BalticSNedeljkovicDTodorovicNRanisavljevMKorovljevDCvejicJ. The impact of six-week dihydrogen-pyrroloquinoline quinone supplementation on mitochondrial biomarkers, brain metabolism, and cognition in elderly individuals with mild cognitive impairment: A randomized controlled trial. J Nutr Health Aging. (2024) 28:100287. doi: 10.1016/j.jnha.2024.100287, PMID: 38908296

[57] Shiojima Y Takahashi M Takahashi R Moriyama H Bagchi D Bagchi M. Effect of dietary pyrroloquinoline quinone disodium salt on cognitive function in healthy volunteers: A randomized, double-blind, placebo-controlled, parallel-group study. J Am Nutr Assoc. (2022) 41:796–809. doi: 10.1080/07315724.2021.1962770, PMID: 34415830

[58] Tamakoshi M Suzuki T Nishihara E Nakamura S Ikemoto K. Pyrroloquinoline quinone disodium salt improves brain function in both younger and older adults. Food Funct. (2023) 14:2496–501. doi: 10.1039/d2fo01515c, PMID: 36807425

[59] Nakano M Murayama Y Hu L Ikemoto K Uetake T Sakatani K. Effects of antioxidant supplements (Biopqq) on cerebral blood flow and oxygen metabolism in the prefrontal cortex. Adv Exp Med Biol. (2016) 923:215–22. doi: 10.1007/978-3-319-38810-6_29, PMID: 27526146

[60] Nakano M Kawasaki Y Suzuki N Takara T. Effects of pyrroloquinoline quinone disodium salt intake on the serum cholesterol levels of healthy Japanese adults. J Nutr Sci Vitaminol (Tokyo). (2015) 61:233–40. doi: 10.3177/jnsv.61.233, PMID: 26226960

[61] Hwang PS Machek SB Cardaci TD Wilburn DT Kim CS Suezaki ES. Effects of pyrroloquinoline quinone (Pqq) supplementation on aerobic exercise performance and indices of mitochondrial biogenesis in untrained men. J Am Coll Nutr. (2020) 39:547–56. doi: 10.1080/07315724.2019.1705203, PMID: 31860387

[62] Harris CB Chowanadisai W Mishchuk DO Satre MA Slupsky CM Rucker RB. Dietary pyrroloquinoline quinone (Pqq) alters indicators of inflammation and mitochondrial-related metabolism in human subjects. J Nutr Biochem. (2013) 24:2076–84. doi: 10.1016/j.jnutbio.2013.07.008, PMID: 24231099

[63] Mattern J Gemmell A Allen PE Mathers KE Regnault TRH Stansfield BK. Oral pyrroloquinoline quinone (Pqq) during pregnancy increases cardiomyocyte endowment in spontaneous iugr Guinea pigs. J Dev Orig Health Dis. (2023) 14:321–4. doi: 10.1017/S2040174423000053, PMID: 36861270 PMC10202840

[64] Mandala A Dobrinskikh E Janssen RC Fiehn O D’Alessandro A Friedman JE. Maternal pyrroloquinoline quinone supplementation improves offspring liver bioactive lipid profiles throughout the lifespan and protects against the development of adult nafld. Int J Mol Sci. (2022) 23:6043. doi: 10.3390/ijms23116043, PMID: 35682720 PMC9181499

[65] Jonscher KR Stewart MS Alfonso-Garcia A DeFelice BC Wang XX Luo Y. Early pqq supplementation has persistent long-term protective effects on developmental programming of hepatic lipotoxicity and inflammation in obese mice. FASEB J. (2017) 31:1434–48. doi: 10.1096/fj.201600906R, PMID: 28007783 PMC5349805

[66] Wang H Li M Chen P Shi X. Anti-inflammatory and antioxidant effects of pyrroloquinoline quinone in L-name-induced preeclampsia-like rat model. Reprod Sci. (2022) 29:578–85. doi: 10.1007/s43032-021-00743-8, PMID: 34542890

[67] Peng Y Xu D Ding Y Zhou X. Supplementation of pqq from pregnancy prevents mk-801-induced schizophrenia-like behaviors in mice. Psychopharmacol (Berl). (2022) 239:2263–75. doi: 10.1007/s00213-022-06113-9, PMID: 35294602

[68] Wang C Zhang B Zhang H Yang W Meng Q Shi B. Effect of dietary pyrroloquinoline quinone disodium in sows on intestinal health of the offspring. Food Funct. (2020) 11:7804–16. doi: 10.1039/d0fo01403f, PMID: 32808626

[69] Zhang B Yang W Zhang H He S Meng Q Chen Z. Effect of pyrroloquinoline quinone disodium in female rats during gestating and lactating on reproductive performance and the intestinal barrier functions in the progeny. Br J Nutr. (2019) 121:818–30. doi: 10.1017/S0007114519000047, PMID: 30688182

[70] Labib MM Alqahtani AM Abo Nahas HH Aldossari RM Almiman BF Ayman Alnumaani S. Novel insights into the antimicrobial and antibiofilm activity of pyrroloquinoline quinone (Pqq); *in vitro*, in silico, and shotgun proteomic studies. Biomolecules. (2024) 14:1018. doi: 10.3390/biom14081018, PMID: 39199405 PMC11352295

