# Supplementary material for: Pre-conditioning with PQQ during pregnancy alleviates LPS-induced placental damage and improves the fetal survival and growth in mice
Source: Front Endocrinol (Lausanne). 2025 Jul 10;16:1617026. doi: 10.3389/fendo.2025.1617026 (PMC12286791; doi:10.3389/fendo.2025.1617026)
Supplement: Supplementary file 1 [file DataSheet1.docx]

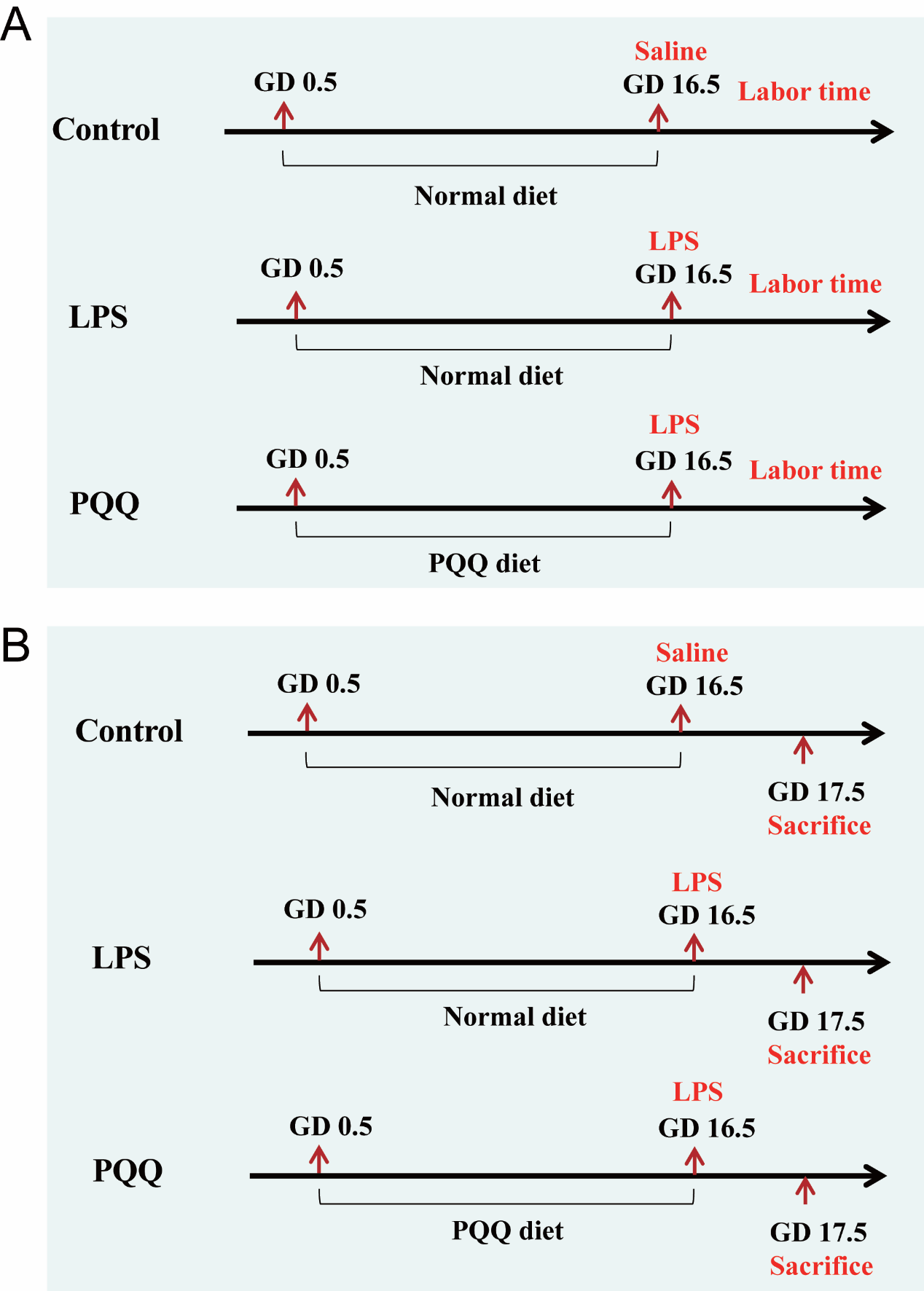


**Supplementary Figure 1. Study design of the present study.**

Pregnant mice at gestational day (GD) 0.5 were divided into three groups: Control group: fed a normal diet; LPS group: fed a normal diet; PQQ group: fed a PQQ-supplemented diet (5 mg/kg diet). At GD 16.5, pregnant mice in the LPS and PQQ groups received LPS treatment. (A) Labor time was monitored. (B) At GD 17.5, pregnant mice were sacrificed for further analysis.


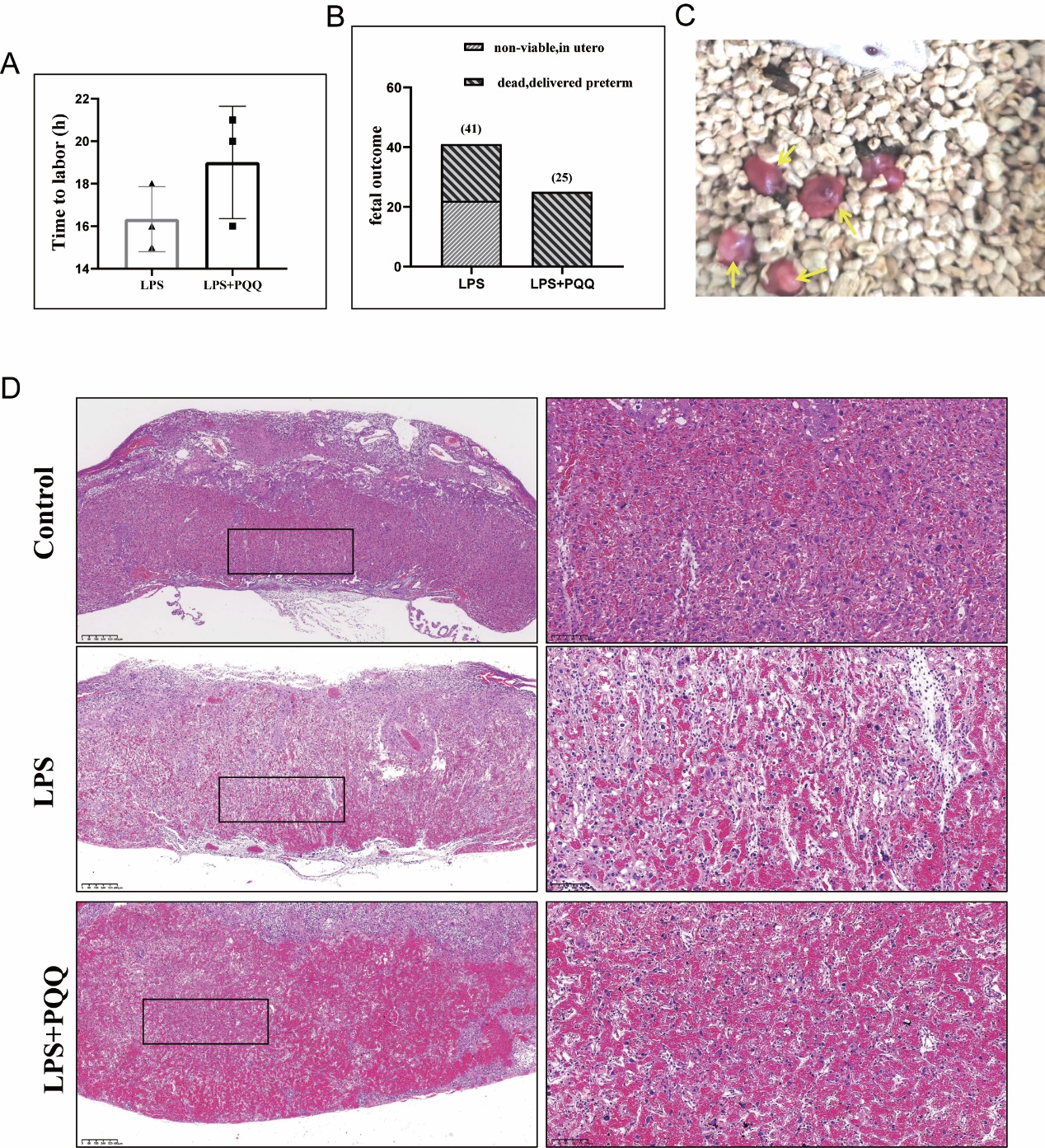


Supplementary Figure 2. PQQ did not provide protective effects with a high dose of LPS.

(A) Mice received either normal saline or LPS (6 µg/mouse), and the time to labor after treatment was recorded. N=3 pregnant mice from each group. (B) Twenty-four hours post-treatment, for mice that had delivered, the number of surviving and deceased fetuses was recorded. For still-pregnant mice, the animals were sacrificed, and fetuses were collected to assess survival in utero. N=4 pregnant mice for LPS group and N=3 pregnant mice for PQQ group. (C) Preterm fetuses delivered enclosed within intact amniotic sacs. The yellow arrow indicates a fetus contained within an unruptured amniotic sac. (D) Twenty-four hours post-treatment, placentas were collected and subjected to hematoxylin and eosin staining to display the placental structure. N=2 placentas from 2 pregnant mice for each group. The area within the black rectangle is magnified in the corresponding images on the right.
